# 

**Published:** 1879-08

**Authors:** 


HERMAN MYNTER, M. D.,

LUCIEN HOWE, M. D.

(Diseases of the Eye and Ear.)

### CONTENTS:

PAGE.

ORIGINAL COMMUNICATIONS:

Spectrum Analysis of Blood, by W. H. Pitt, M. D., . . . . . . . . . 1
Use of Permanganate of Potassa in Chronic Otorrhœa, by Lucien Howe, M. D., . 6
Newgrowth in the Bladder, by Herman Mynter, M. D., . . . . . . 9

TRANSLATIONS:

"Is the Continued Use of a Small Quantity of Salicylic Acid Detrimental to Health?" by E. V. Stoddard, M. D., . . . . 14

SELECTIONS:

Cholera Infantum, by Preston B. Scott, M. D., 17
Œsophagotomy, . . . . . . . . 22
Carbolic Acid for the Cure of Internal Piles, 24
The Sphygmograph for Diagnosis, Peculiarly Slow Pulse, . . . . . . . 24
The Medical Expert System in Germany, . 25
Retention of the Urine, . . . . . . 26
Salicylic Acid against Tænia, . . . 27
Solvents of Iodoform, . . . . . . 27
Whooping Cough, . . . . . . . . 28
To get Leeches to Fasten, . . . . . 28
External use of Digitalis in Suppression of Urine, . . . . . . . . 29
Salicylate of Soda in Rheumatism, . . 29
Syrup of Dover's Powder, . . . . . 29
An Account of the Perineosinuexereeinator, 30
Partial Dislocation of the Fourth Cervical Vertebra due to Muscular Action, by John A. Wyett, M. D., . . . . . . 34

EDITORIAL, . . . . . . . . 35

A Word for the JOURNAL, by Prof. J. F. Miner, M. D., . . . . . . . 37

REVIEWS, . . . . . . . . . 38

Books and Pamphlets Received, . . . 40

